# Changes on local travel behaviors under travel reduction-related interventions during COVID-19 pandemic: a case study in Hong Kong

**DOI:** 10.1007/s44213-023-00006-z

**Published:** 2023-03-06

**Authors:** Shujia Shang, Wei Jia, Shiyao Zhang, Boni Su, Reynold Cheng, Yuguo Li, Nan Zhang

**Affiliations:** 1 Beijing Key Laboratory of Green Built Environment and Energy Efficient Technology, Beijing University of Technology, Beijing, China; 2 Department of Mechanical Engineering, The University of Hong Kong, Hong Kong, China; 3 The Research Institute for Trustworthy Autonomous Systems, Southern University of Science and Technology, Shenzhen, 518055 China; 4 China Electric Power Planning & Engineering Institute, Beijing, China; 5 Department of Computer Science, The University of Hong Kong, Hong Kong, SAR China; 6 School of Public Health, Li Ka Shing Faculty of Medicine, University of Hong Kong, Hong Kong, SAR China

**Keywords:** COVID-19, Public transport, Social distance, Human behavior, Subway

## Abstract

The emerging Omicron variant poses a serious threat to human health. Public transports play a critical role in infection spread. Based on the data of nearly 4 billion smartcard uses, between January 1, 2019 and January 31, 2021 from the Mass Transit Railway Corporation of Hong Kong, we analyzed the subway travel behavior of different population groups (adults, children, students and senior citizens) due to the COVID-19 pandemic and human travel behavior under different interventions (e.g. work suspension, school closure). Due to the pandemic, the number of MTR passengers (the daily number of passengers in close proximity in subway carriages) decreased by 37.4% (40.8%) for adults, 80.3% (78.5%) for children, 71.6% (71.6%) for students, and 33.5% (36.1%) for senior citizens. Due to work from home (school suspension), the number of contacted adults (students/children) in the same carriage during the rush hours decreased by 39.6% (38.6%/43.2%). If all workers, students, and children were encouraged to commute avoiding rush hours, the possible repeated contacts during rush hour of adults, children and students decreased by 73.3%, 77.9% and 79.5%, respectively. Since adults accounted for 87.3% of the total number of subway passengers during the pandemic, work from home and staggered shift pattern of workers can reduce the infection risk effectively. Our objective is to find the changes of local travel behavior due to the pandemic. From the perspective of public transports, the results provide a scientific support for COVID-19 prevention and control in cities.

**Supplementary Information:**

The online version contains supplementary material available at 10.1007/s44213-023-00006-z.

## Introduction

Since first emerging in the end of 2019, COVID-19 has been threatening human lives and societies. As the Omicron variant first appeared at the end of 2021 (Del Rio et al., 2022), it spread rapidly around the globe. By the end of October 2022, more than 623 million infections and 6.6 million deaths had been reported globally (WHO 2022). The Omicron variant is highly infectious with a basic reproductive number *R*_*0*_ of 4.5–10, which is several times that of the Delta variant (Baker et al. 2021; Halamicek et al. 2022; Davido et al. 2022). As one of the cities with the highest population density, Hong Kong had a total of 2.4 million (32% of the total population) confirmed COVID-19 cases by the end of December 2022 (NHKG 2022a). Of the 10,000 plus reported cases after February 2022, more than 80% of these cases were infected with the Omicron variant (NHKG 2022b). Due to the high infectivity of the Omicron variant, interventions which had been effective for prevention and control of previous variants (e.g. Delta) were unable to meet the current needs, and a serious outbreak was triggered in Hong Kong.

Close contact (short-range airborne and large droplet), long-range airborne and fomite transmission are the three potential transmission routes for SARS-CoV-2 (Lotfi et al. 2020; Rahman et al. 2020). Many studies have shown that close contact is the dominant route of COVID-19 transmission (Karia et al. 2020; Zhao et al. 2020), Maintaining a social distance of 1.5 m is considered to be the most effective intervention for most respiratory infectious diseases including COVID-19 transmission (Qian and Jiang 2022; Sun and Zhai 2020; Aquino et al. 2020). and researchers have emphasized the importance of social distancing in disease prevention through modeling and simulation (Chinazzi et al. 2020). However, assessing people’s travel behavior is an important way to quantify social distancing (Anderson et al. 2020). Many countries and cities have implemented social distancing measures such as work suspension (Ruiz-Frutos et al. 2021), school closure (Zhang et al. 2020) and limits on bars and restaurants (Abouk and Heydari 2021).

Public transport is important for the local travel needs of residents. It not only provides a venue for infection spread, but also connects people from different regions (Zheng et al. 2020). Therefore, prevention and control of COVID-19 on public transport is particularly important. Taking influenza as an example, without mask wearing, about 4% of infections occur during travel on subways (Cooley et al. 2011). Therefore, for public transportation using the subway as an example, it is very important to formulate effective epidemic prevention and control strategies according to the travel behavior of passengers. Especially because of the omicron variant with a high basic reproductive number of R_0_ pose a great risk of transmission.

Based on data analysis and simulations, many studies have proposed interventions for COVID-19 prevention and control on public transport (Ozdemir et al. 2022; Shen et al. 2020). However, these studies had a number of shortcomings. Firstly, individual differences were often ignored (Zhang et al. 2021a), for example, susceptibility to viruses (Vakili et al. 2020), as well as transport system usage, differ according to age. Secondly, due to the lack of data concerning real human close contact behaviors, it is difficult to accurately evaluate close contact transmission in subways (Nissen et al. 2020). Finally, many studies did not base their results on real public transport operational data, and therefore cannot propose accurate strategies for COVID-19 prevention and control (Guan et al. 2020).

In Hong Kong, the subway, known as the Mass Transit Railway (MTR), is the most used form of public transport, accounting for more than 40% of local passengers (Zhang et al. 2021b). For this study, we obtained nearly 4 billion smartcard use data for four population groups (adults, children, students and senior citizens) from January 1, 2019 to January 31, 2021. Taking the four waves of COVID-19 outbreaks during this period as examples, we analyzed changes in local travel behavior due to these pandemic waves. Based on the real people’s travel behavior before the pandemic, different interventions (work from home, school suspension, staggered shift travel pattern, and reduction on subway riding) are proposed to ensure social distance, and by simulating the behavior of people under different epidemic prevention measures. This provides scientific support for strategies to deploy for COVID-19 prevention and control in the subway system.

## Method

### Data sources

Nearly 4 billion smartcard use data from January 1, 2019 to January 31, 2021 was obtained from the Mass Transit Railway Corporation (MTRC) of Hong Kong. This data included entry and exit station, the entry and exit time at the second level, the type of smartcard, etc. All transport system users were divided into four categories based on the card type: child (aged between 3 and 11), student (aged between 12 and 25 enrolled in primary/secondary/high school, university, or higher education institution), adult (aged between 19 and 65 excluding students), and senior citizen (aged over 65 years). In addition, pandemic-related data (e.g. daily number of confirmed cases) was obtained from the Center for Health Protection of Hong Kong (HKCHP 2021). The detailed data of smartcard swiping is shown in Table 1.

**Table 1:** Detailed Hong Kong MTR swiping original data

Card ID	Date	Time for enter	Time for leave	Enter station	Leave station	Ticket type
9******1	2019/3/26	7:18:50	7:36:28	Mong Kok	Diamond Hill	Adult
9******2	2019/3/26	7:14:24	7:30:27	Center	Causeway Bay	Child
9******3	2019/3/26	7:04:44	7:27:00	Kwai Hing	Kowloon	Student
9******4	2019/3/26	7:09:46	7:44:50	University	Lam Tin	Senior

### Study area

This study aims to reduces the interpersonal contacts by changing travel behaviors to reduce the infection risk in subways. A typical subway train in Hong Kong consists of eight carriages, and each carriage is 22 m long and 3.2 m wide (Baidu, 2022).

Due to the huge difference on local travel behavior between weekdays and weekends, local travel behavior and efficiency assessment for interventions for both weekdays and weekends were analyzed. Considering that many workers in Hong Kong need to work on Saturday, in this study, only Sunday was regarded as weekend.

Rush hours: Rush hours are the times of the day when the number of passengers in the carriage reach the peak. The rush hours for adults (Children/students) were 7:30–9:00 and 18:00–19:30 (7:00 to 8:00 and 15:30 to 16:30).

Non-rush hours: Train operation period except rush hours.

There were four waves of COVID-19 outbreaks in Hong Kong between January 1, 2020 and January 31, 2021. During each wave, we denoted the week with the highest total number of infections to be the pandemic week. Therefore, there were four pandemic weeks during our study period, the weekly number of reported confirmed cases is shown in Fig. S1. Detailed daily pandemic data from January 23, 2020 to May 31, 2021 is shown in Fig. S2. The four pandemic weeks covered in this study were: Mar. 26 to Apr. 1, 2020; Jul. 24 to 30, 2020; Dec. 4 to 10, 2020; Jan. 18 to 24, 2021. To analyze changes in local travel behaviors, we obtained data from 4 weeks in 2019 corresponding to the same periods as the pandemic weeks of 2020 and 2021, to act as control groups (Mar. 26 to Apr. 1, 2019; Jul. 24 to 30, 2019; Dec. 4 to 10, 2019; Jan. 18 to 24, 2019). The above smartcard swipe data was collected and analyzed, to determine: how local travel behavior had changed due to the pandemic and how non-pharmaceutical interventions (e.g. work from home, school suspension, staggered shift travel pattern, and travel reduction) influenced interpersonal contacts in subways.

### Data processing

Not all smartcard data was valid to use, and the following three screening methods were used to screen the raw data.

1. The swiping record of smartcard for both enter and leave the station should exist simultaneously.
2. The entry and exit stations of a travel should be different.
3. The time of smartcard swiping should be within the subway’s operation time.

After the above data screening, nearly 4 billion card swiping data from were obtained, and less than 5% of them were invalid.

Hong Kong Metro had 10 lines (excluding Light Rail and High Speed Rail) and 98 heavy rail stations (MTR of Hong Kong, 2022) (Fig. S3). We utilized Dijkstra’s algorithm to generate the shortest path from any station A to any station B for MTR, Hong Kong. The shortest distance is defined as the minimum number of boarding stations, which includes the actual boarding stations and the equivalent stations for the inter-line transfer. The common inter-line transfer is considered as one station interval except for a few special inter-line transfers, that is, 3 stations for the transfer between Central and Hong Kong, 4 stations for the transfer between Kowloon and Austin, 5 stations for the transfer between East Tsim Sha Tsui and Tsim Sha Tsui and the transfer between Kowloon and Tsing Yi. We also obtained a weekly schedule for each train in each MTR line from the MTR website and Google map, which means that each train has its own number. And then we allocated each passenger to the corresponding train based on the following two principles. The first principle is to allocate each passenger an optimal route, including the boarding lines and stations, using the enter/leave stations of each passenger and the shortest path determined by Dijkstra’s algorithm. The second principle is to allocate each passenger to the corresponding train on the shortest path based on the enter/leave station time and the weekly schedule of each train. The passenger should arrive at the station platform before the arrival time of each train. Note that we also consider a 2-min walk from the station gate to the station platform, a 2-min walk for the common inter-line transfer and a 5-min walk for the special inter-line transfer. Finally, we allocated 1.5 billion passengers to the corresponding MTR train in Hong Kong successfully.

In this study, travel reduction-related interventions for COVID-19 prevention and control in subways including work from home (or AB work shift), class suspension, staggered shift travel pattern, and travel reduction were considered. Since the local travel behavior before the pandemic without any interventions represented the real condition, the efficiency of interventions in subways were analyzed based on normal local travel behaviors during the non-pandemic weeks. All interventions for COVID-19 prevention and control in subways considered in this study are introduced in Supplementary Information.

## Result

### Changes on local travel behavior due to the pandemic

Due to the pandemic, the total number of MTR passengers decreased by an average of 41.0% (37.4%, 80.3%, 71.6% and 33.5% for adults, children, students and senior citizens, respectively). During four non-pandemic weeks, 78.9% (*n* = 1.9 million), 3.5% (*n* = 0.08 million), 7.3% (*n* = 0.18 million), and 10.2% (*n* = 0.25 million) of MTR passengers used adult, child, student and senior citizen cards, respectively. Where during four pandemic weeks, 83.7% (*n* = 1.2 million), 1.2% (*n* = 0.02 million), 3.5% (*n* = 0.05 million), and 11.6% (*n* = 0.17 million) of MTR passengers used adult, child, student and senior citizen cards, respectively.

**Fig. 1:**
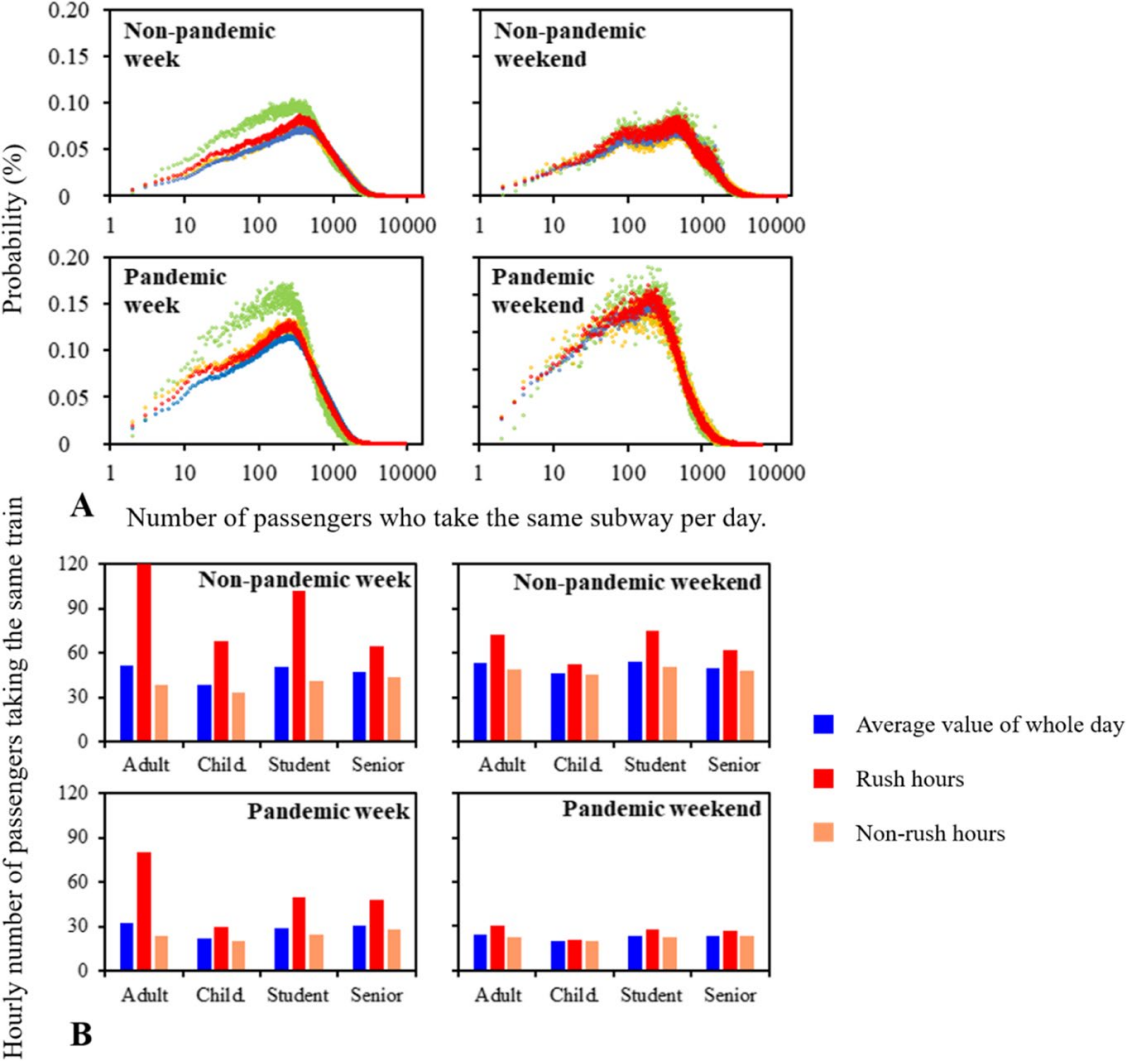
Subway riding behavior in the same train during weekdays and weekends before and during the pandemic by population group. **A** Probability distribution of daily number of passengers on the same train (*DPST*); **B** Hourly number of passengers taking the same train (*HPST*)

Although the number of passengers decreased significantly, the daily number of subway trains remained unchanged (Fig. S4). Figure 1A shows the probability distribution of the number of passengers in the subway. Due to the pandemic, daily number of passengers on the same train (*DPST*) of adults, children, students and senior citizens during weekdays (weekends) was decreased by 37.2% (52.2%), 41.3% (50.7%), 43.4% (53.7%), and 33.5% (49.8%), respectively. Children had the lowest *DPST* of 637, while adults had the highest of 792.

During the same period during a day, *DPST* changes little for all population groups (Fig. 1B). In the weekday before (during) the pandemic, the ratio of the hourly number of passengers taking the same train (*HPST*) of whole day, rush hours and non-rush hours was 1: 1.9(1.8): 0.8(0.8). In the weekend before (during) the pandemic, the ratio of the hourly number of passengers taking the same train (*HPST*) of whole day, rush hours and non-rush hours was 1: 1.3(1.8): 0.9.

In rush hours of the weekday before the pandemic, there were significant differences in *HPST* among four population groups (*p* < 0.05). The *HPST* for adults, children, students and senior citizens were 120.3, 68.3, 102.0 and 64.9, respectively. Adults and students have significantly higher *HPST* than children and senior citizens because they have to go to work and school.

The distribution on contact time of four population groups was different (Fig. S5). In pandemic (non-pandemic) weekday, the average daily duration on the same train of adults, children, students and senior citizens on weekends was 4.0 (4.0), 4.0 (3.8), 4.0 (3.8) and 4.1 (4.3) minutes, respectively, which were 8.4% (5.6%), 6.9% (8.0%), 9.9% (6.1%) and 4.6% (3.4%) higher than them on weekday, respectively.

A person may take the MTR with the same passenger many times per day. The probability distribution of daily number of passengers on the same train showed a monotonically logarithmic decrease (Fig. S6). More than 99% of possible daily repeated contacts in the same train (*DRC*) were only once. The number of *DRC* during the non-pandemic weekdays was 1.6 times higher than it during the pandemic weekdays. Due to the pandemic, *DRC* decreased significantly (*p* < 0.01), the average number of *DRC* of adults, children, students and senior citizens during weekdays (weekends) decreased by 66.8% (75.4%), 94.7% (83.8%), 88.6% (83.3%), and 59.5% (73.4%), respectively. Adults had the highest number of *DRC* with others (the detailed distribution is shown in Fig. S7).

**Fig. 2:**
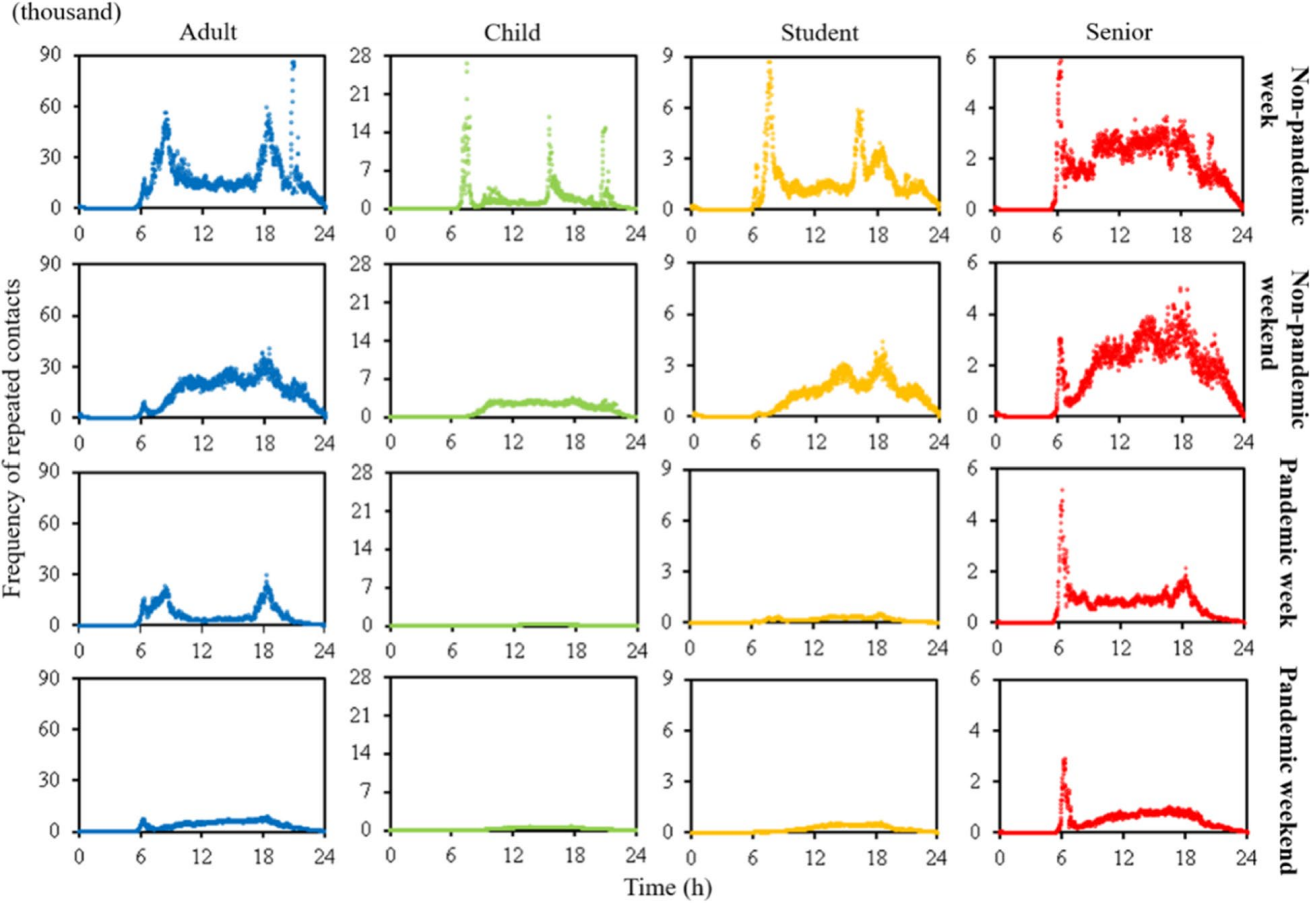
Time-variant frequency of possible repeated contact on the same train of four population groups

The frequency of possible repeated contacts on the same train (*FRC*) in subways changes significantly with time and population group (Fig. 2). Before the pandemic, the rush hours for adults were 7:30–9:00 and 18:00–19:30. Due to fixed residential area and work places, many workers had a high *FRC* during the morning and evening hours, up to 60,000. During pandemic weekdays, there was still two significant rush hours because work from home was not implemented for all companies. Due to the pandemic, *FRC* of adults in the rush hours (non-peak hours) decreased by 58.1% (70.8%).

Children/students has an earlier and shorter rush hour than adults (7:00 to 8:00 and 15:30 to 16:30). Due to the pandemic, *FRC* of children/students was reduced by 97.8% (92.6%) during rush (non-rush) hours. Before the pandemic, comparing with weekdays, children and students reduced their *FRC* during weekends by 7.6% and 19.4%, respectively. However, due to class suspension, during the pandemic, *FRC* of children and students during weekends were increased by 182.3% and 18.5%, respectively. The pandemic influenced on travel behaviors, especially for children and students. Between 20:30 and 21:00 during non-pandemic weekdays, adults and children had a much higher peak of *FRC* than it during both morning and evening rush hours. However, due to the pandemic, the *FRC* for adults and children was decreased by 92.0% and 99.0% during this period, respectively. This showed that the pandemic had a significant impact on non-essential travel.

The senior citizen had the minimal difference (3.2%) of *FRC* between weekdays and weekends before the pandemic. However, due to the pandemic, the *FRC* of senior citizens in weekends was 32.1% lower than it in weekdays.

**Fig. 3:**
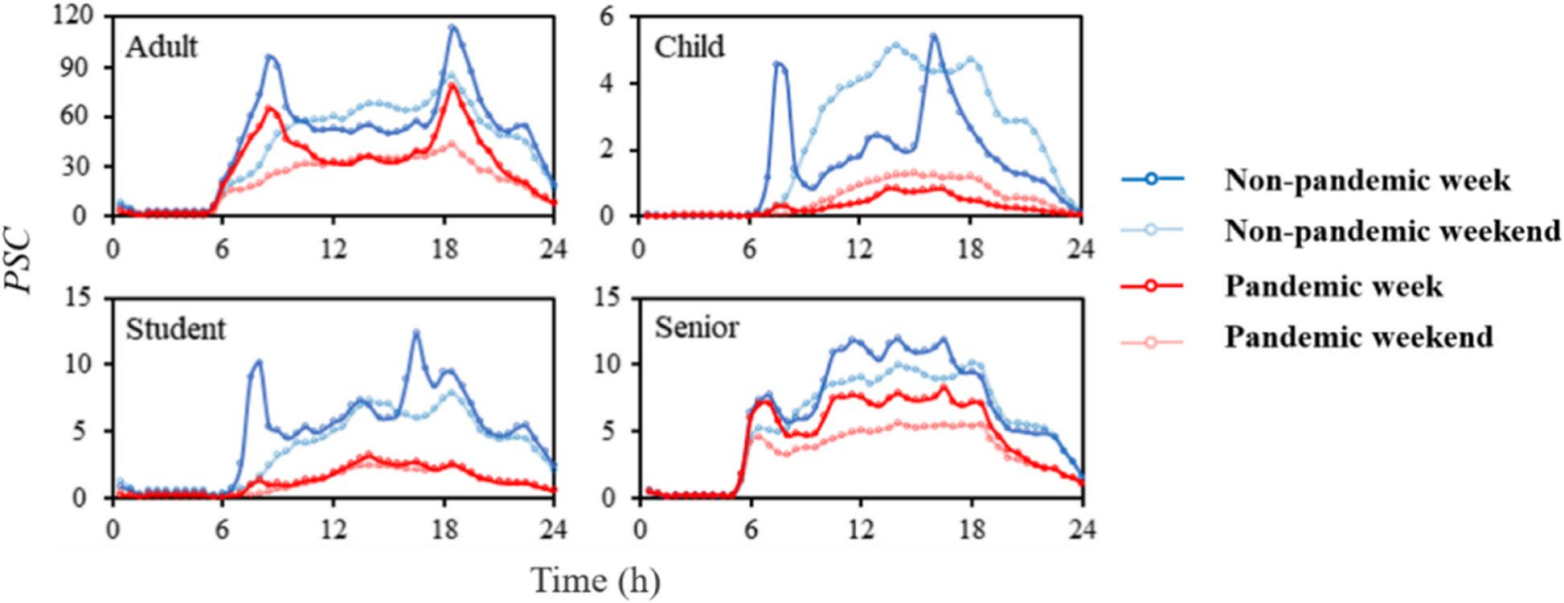
Number of passengers on the same carriage (*PSC*) of four population groups

Due to the pandemic, the number of passengers on the same carriage (*PSC*) of adults, children, students and senior citizens during weekdays (weekends) decreased by 34.5% (47.8%), 80.0% (78.3%), 72.9% (70.3%), and 30.2% (42.9%), respectively (Fig. 3). During the rush hour of pandemic weekdays, the PSC of adults, children and students decreased by 32.6%, 88.1% and 81.4%, respectively. Comparing with the weekdays, the *PSC* of adults, students, and senior citizens in pandemic (non-pandemic) weekends was decreased by 9.6% (28.0%), 22.9% (15.4%), and 11.1% (27.3%), respectively. However, children had 55.3% (66.7%) more *PSC* during pandemic (non-pandemic) weekends than during weekdays. During the non-pandemic weekdays, there averagely were 94 adults, 5 children, and 10 students in a carriage. Compared with non-pandemic period, the reduction of *PSC* of senior citizens during the pandemic was the smallest.

**Fig. 4:**
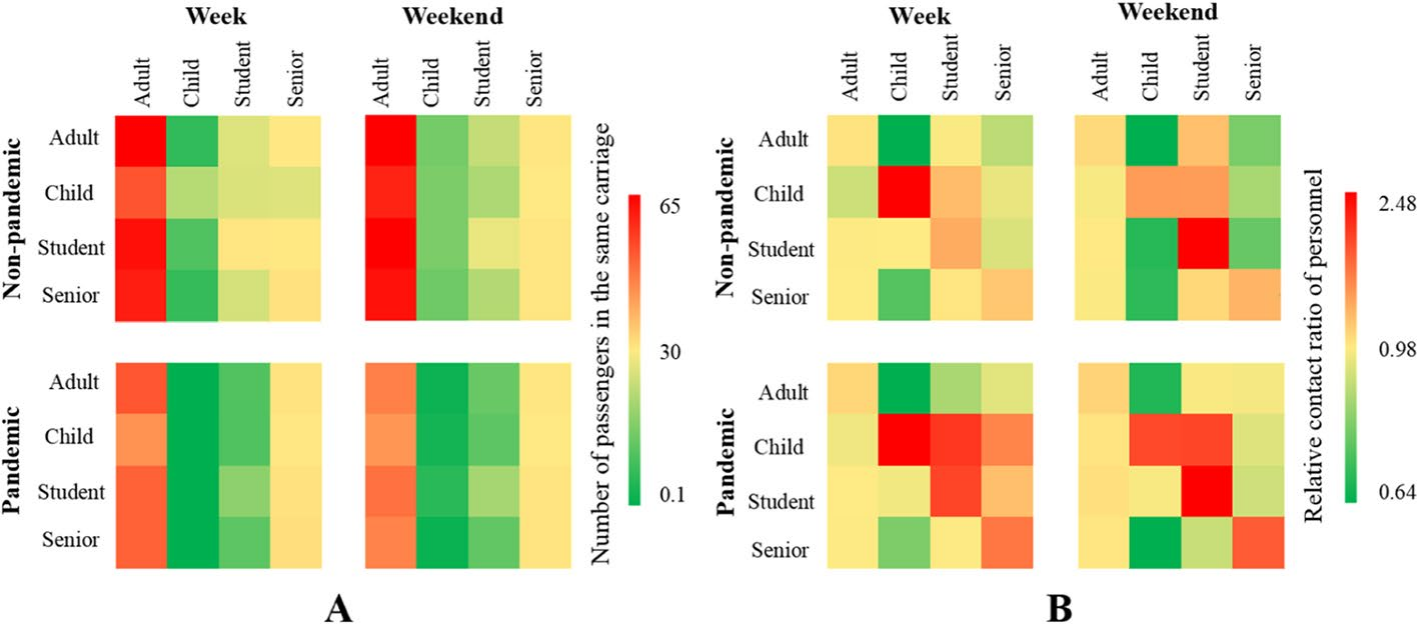
Contact between different population group. **A** Contact matrix; **B** relative contact matrix

Due to the pandemic, number of passengers in the same carriage (*PSC*) was reduced during weekdays for all population groups, especially for children (Fig. 4A). Adults, children, students, and senior citizens reduced *PSC* with children by 81.6%, 90.3%, 81.6% and 78.9%, respectively. Considering the proportion of each population group, passengers tended to have contacts with same-type passengers (Fig. 4B). During pandemic weekdays, contacts between children and senior citizens increased significantly, while during pandemic weekends, adults had much more contacts with senior citizens.

Compared with the rush hours before (during) the pandemic, the number of contacts between adults, children, students, senior citizens and adults in the same carriage decreased by 22.9% (25.8%), 21.1% (26.8%), 26.7% (32.4%) and 21.4% (24.5%), respectively. The number of contacts between adults, students, senior citizens and children in the same carriage increased in weekends compared in weekdays. Compared to the weekend, the number of adults, students and senior citizens contacted with children in the same carriage in weekdays before (during) the pandemic increased by 87.7% (105.4%), 48.1% (74.9%) and 81.0% (79.3%), respectively. However, the contacts between children in the same carriage decreased by 25.3% due to school suspension.

All population groups tended to contact with same-type passengers during both rush and non-rush hours. During the rush hours of the weekend, the contact between the four groups and senior citizens in the same carriage was significantly reduced, which indicate that senior citizens avoided unnecessary travel during rush hours during the pandemic. During the rush hours of the weekend before the pandemic, contacts between all population groups and students increased sharply comparing with it during the non-rush hours. The detailed distribution was shown in Figs. S8 and S9.

### Travel reduction-related interventions

Based on the normal travel behaviors before the pandemic period, this section analyzed how interventions including work from home, school suspension, staggered shifts travel pattern and reduction on subway riding of different population group influence the interpersonal contacts in subways.

#### Work from home and school suspension

Work from home and school suspension can significantly reduce interpersonal contacts of adults, children and students (Fig. S10). Due to the work from home (school suspension), the number of passengers in the same carriage (*PSC*) of adults (students/children) during their rush hours decreased by 39.6% (38.6%/43.2%). Due to more passengers were adults, work from home in weekday can significantly reduce the *PSC* of all population groups in the rush hours.

**Fig. 5:**
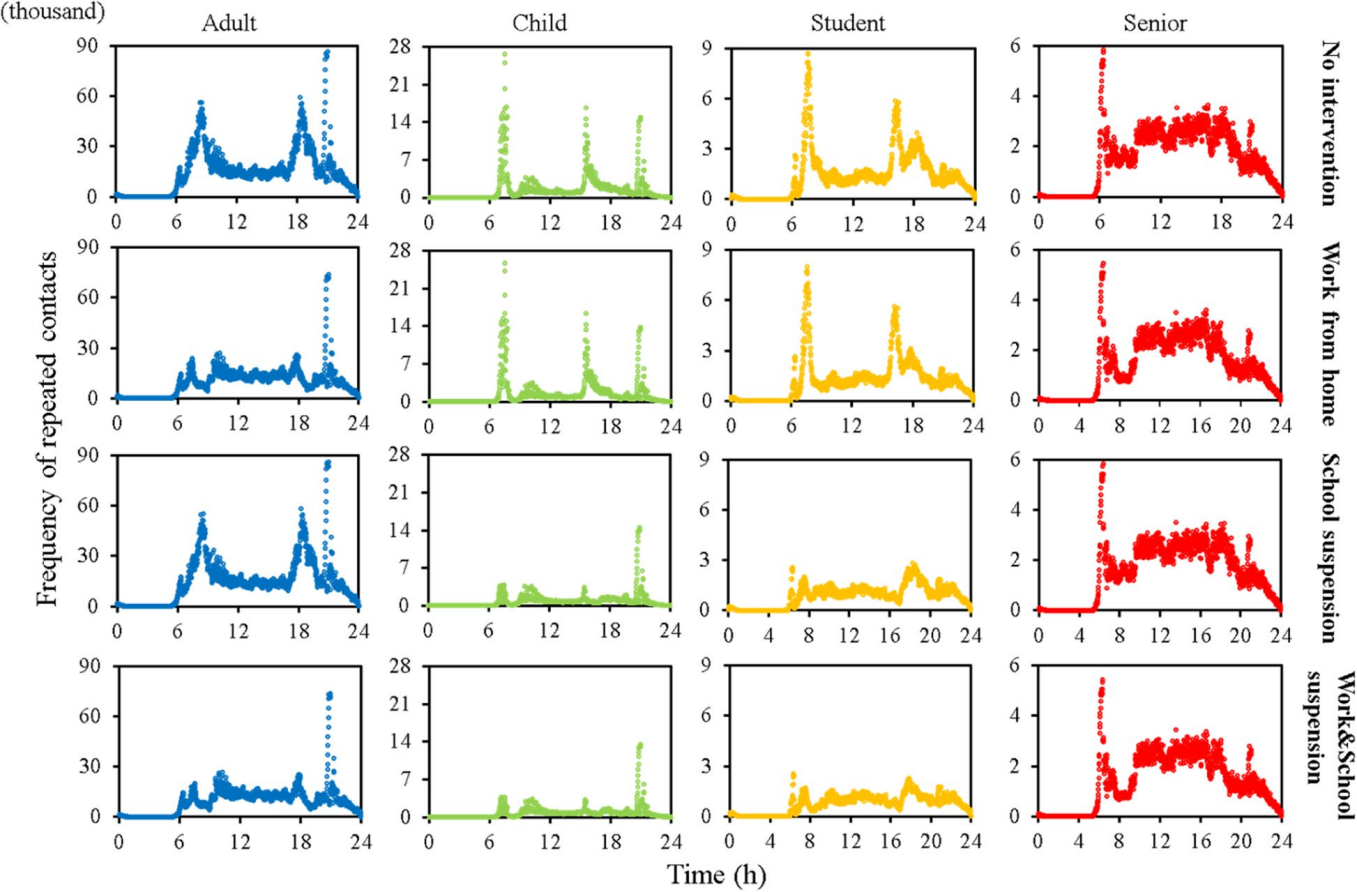
Frequency of possible repeated contacts on the same train (*FRC*) by time of four population groups under work from home, school suspension, and both work and school suspension

From Fig. 5, when work from home, school suspension, and both work and school suspension were implemented, the *FRC* of adults during the rush hours (7:30–9:00 and 18:00–19:30) were reduced by 76.3%, 2.9% and 77.8%, respectively, and the *FRC* of students (children) during the rush hours (7:00–8:00 and 15:30–16:30) was reduced by 8.7% (4.6%), 76.0% (81.3%) and 79.5% (82.6%), respectively.

If both work and school suspension were implemented, the *FRC* of adults, children, students and senior citizens would be decreased by 37.8%, 50.2%, 45.3% and 11.8%, respectively. If only work from home was implemented, the average *FRC* of adults, children, students and senior citizens would be decreased by 35.9%, 8.2%, 13.4% and 9.8%, respectively.

If both work and school suspension were implemented, the daily number of passengers in the same train (*DPST*) of adults, children, students and senior citizens decreased by 21.4%, 23.7%, 23.0% and 9.7%, respectively, and children had the lowest *DPST* of 647. The detailed distribution is shown in Fig. S11.

#### Staggered shifts travel pattern

**Fig. 6:**
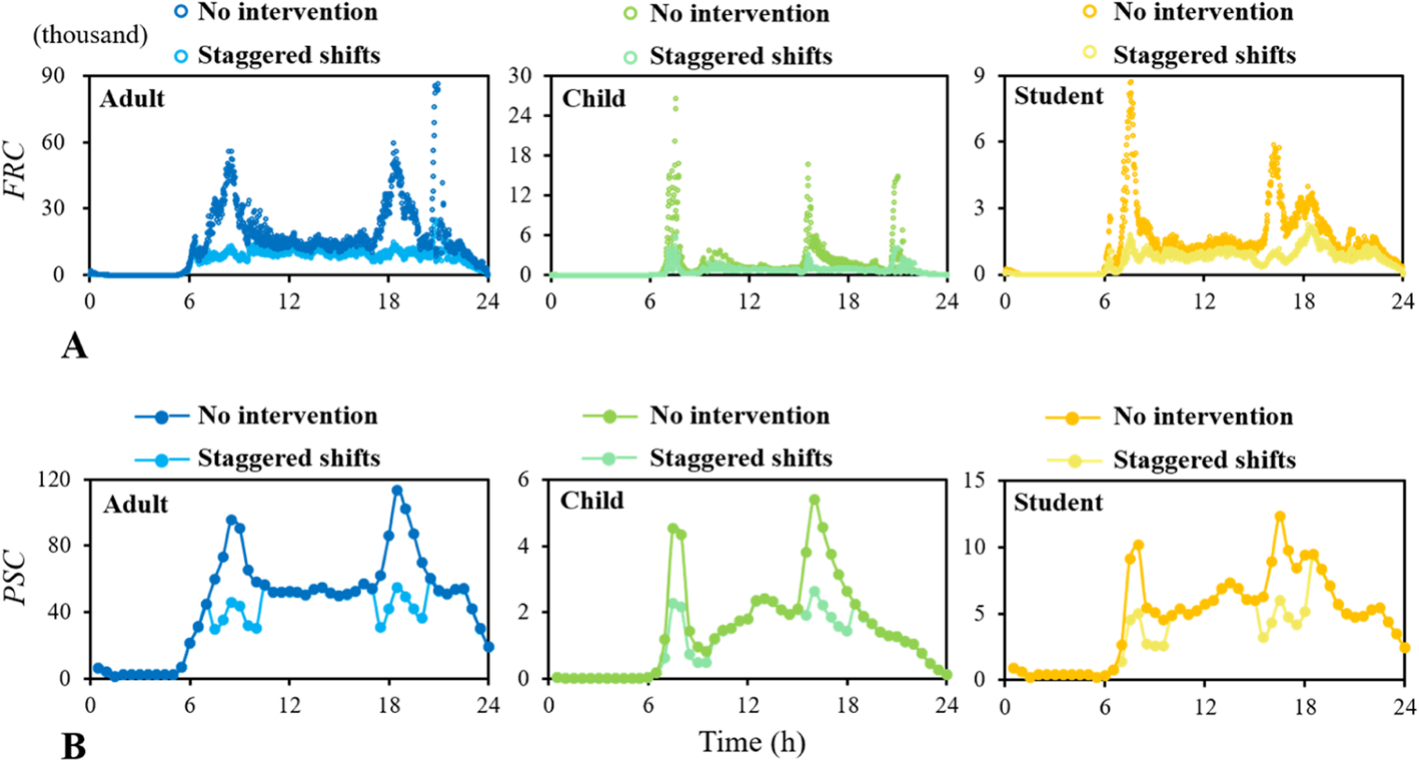
Time-variant distribution with (**A**) frequency of possible repeated contacts on the same train (*FRC*) and (**B**) number of passengers in the same carriage (*PSC*) of adults, children, and students under staggered shift travel pattern

When the staggered shift travel patterns of workers, students, and children were implemented, the *FRC* of workers, students, and children during their rush hours were reduced by 73.3%, 79.5%, and 77.9%, respectively (Fig. 6A). The number of passengers in the same carriage (*PSC*) of children and students during the rush hours (single day) of school was reduced by 50.7% (25.6%) and 50.9% (20.2%), respectively, and the *PSC* of adults during the rush hours (single day) was reduced by 51.7%. During the rush hours, the number of adults, children and students in the same carriage was 42.5, 2.3 and 5.0 respectively (Fig. 6B).

#### Travel reduction of different population groups

If the travel of adults was reduced by 90% during the weekday (weekend) before the pandemic, the *DPST* would be decrease by 84.8% (83.6%) for adults, 65.3% (70.2%) for children, 68.6% (70.1%) for students, and 69.5% (70.1%) for senior citizens (Fig. S12). If the travel of children, students and senior citizens was reduced by 90%, the *DPST* of children, students and senior citizens would be reduced by 42.5%, 51.3% and 48.0%, respectively.

## Discussion

The COVID-19 has threatened almost all areas with people in the world, especially in large cities with high population density (Chen et al. 2020). Taking Hong Kong as an example, MTR is the main public transport. Local travel behaviors on subway was greatly affected by the COVID-19 pandemic and changes on local travel behaviors also had a significant impact on the spread of COVID-19 (Megahed and Ghoneim 2020; Iacus et al. 2020). Between January 1, 2020 and January 31, 2021, Hong Kong had experienced four pandemic waves. Take MTR data during 2019 as control groups, approaching 4 billion valid card swiping data were collected to analyze the changes on local travel behaviors due to the pandemic. The impact of travel reduction-related interventions (e.g. work from home, school suspension, staggered shift travel pattern, travel reduction) on interpersonal contacts were simulated and analyzed.

Due to the COVID-19 pandemic, people reduced their travel significantly (Jenelius and Cebecauer 2020). International travels were banned in many countries due to the pandemic (Sun et al. 2021). Residents deliberately avoid to take public transports (e.g. subway) and turned to use private cars (Chang et al. 2020). COVID-19 has affected people’s travel behavior in the MTR in Hong Kong severely. We found that, due to the pandemic, Hong Kong adults, children, students and senior citizens reduced their subway riding by 37.2%, 41.3%, 43.4%, and 33.5%, respectively. Subway is one of the most important public transports (Wu and Hong, 2017), especially in Hong Kong. It is important to carry out an effective strategy on COVID-19 prevention and control in subways (Feng et al. 2020).

Most of passengers taking public transports were adults, especially during the rush hours. However, due to the short social distance, the infection risk via close contacts would be very high. During the serious pandemic, half of Europeans worked at home (Galanti et al. 2021), working from home has become a policy priority in many countries (Vyas and Butakhieo 2021). Therefore, the local government should take measures of work from home or off-peak commuting to reduce the infection risk on public transports. Working from home for jobs that require attendance at work (e.g. construction workers, healthcare, agriculture, and hospitality). The policy of working from home can be implemented for some occupations, but this will increase the danger of exposure if employees commute a significant distance (Lo et al. 2011). Work from home was usually taken by adults (Dubey and Tripathi 2020) to avoid taking public transportation, and flexible working hours could also ensure the effective business operations (Purwanto et al. 2020). A fair allocation of adults who work from home can not only lower turnover rates, boost worker productivity (Baker et al. 2007), and ensure that the city’s daily operations run smoothly.

Due to the weak awareness of personal protection and relative short social distance, children and students would tend to be infected (Iachini et al. 2021) if all types of people had the same susceptibility. The Education Bureau will ensure the quality of distant learning (Lau, EYH, Lee K 2021), and children and students should implement mandatory school closure measures, which can considerably protect their safety. However, study showed that children and students have a higher resistance to COVID-19 than adults and senior citizens (Jing et al. 2020), school suspension is not that effective as we expected. In addition, the number of subway travels of children was the smallest among all population groups, which means changes on children’s travel behavior influence the pandemic least. However, school suspension has also been adopted in many areas to relieve the spread of the pandemic (UNESCO 2020). For other respiratory infectious diseases such as influenza, which is highly susceptible for children and students (Smith et al. 2017), school suspension would be very effective (Kao et al. 2012). School suspension reduced the average frequency of possible repeated contacts on the same train (*FRC*) of students and children during rush hours by 76.0% and 81.3%, respectively.

Among four population groups, senior citizens showed the least changes on local travel behavior due to the pandemic, and still maintained a relatively high frequency of subway riding. However, many older people suffered from chronic diseases (e.g. diabetes mellitus), which lead to lower resistance and more complications (Dhama et al. 2020; Csiszar et al. 2020). The susceptibility and mortality rate of COVID-19 are 1.6 and 5.1 times of that of adults, respectively (Jing et al. 2020; Lv et al. 2021). We found that senior citizens had the longest daily exposure time in subways, which led to the greatest risk of exposure in carriages (Lo et al. 2021). Unfortunately, the vaccination rate decreases with age (Thanapluetiwong et al. 2021). In Hong Kong, less than 20% of older care home residents were fully vaccinated against COVID-19 when Omicron came at the end of February 2022 (Ma and Parry 2022). The vaccination rate of people over 60 is only 62% of that of adults (Smith et al. 2022). Hong Kong is a rapidly aging city (Jayantha et al. 2018), therefore, government should prioritize vaccination for senior citizens to reduce their susceptibility and mortality (Monahan et al. 2020). Moreover, it is important to strengthen the awareness of pandemic prevention and control for older people because most of their travel are unnecessary.

In the rush hours of commuting workers (7:30–9:00 and 18:00–19:30) and students (7:00–8:00 and 15:30–16:30), there were high *FRC* because large flow of passengers. Therefore, the government should implement measures that stagger peak travel, such as mandating adults commute at different times during the day; Children and students go to and from school in various grades (kindergarten, elementary school, junior high school and high school). We also found that the highest peak on *FRC* for both adults and children was between 20:30 and 21:00 during weekdays other than the morning and evening rush hours as we expected. The main reason may be that they usually go to shopping centers, entertainments, restaurants, and other places for non-essential activities during this period. Local governments should take relevant interventions such as closing shopping malls earlier and restricting population flow in public indoor environments, to reduce the close contacts during this period. Reducing repeated contacts among people in the social network can greatly reduce the transmission of the virus (Block et al. 2020). This study found that more than 99% of passengers repeated contacts in the same train (*DRC*) were only once. But in the weekend before the pandemic, the *DCR* of adults reached a maximum of 15. No matter what *DRC* is, in all population groups, adults have the highest frequency of contacts. Dispersing the commuting time of workers would be effective for repeated contact reduction (Tirachini and Cats 2020). In addition, increasing the departure frequency of subway trains can also reduce the density of passengers, thus reducing the infection risk.

The infection risk of susceptible population increased significantly with the passenger flow, and the infection risk varies with time (Li et al. 2022). There was a negative correlation between COVID-19 spread and social distance, while short social distance would lead to (Seong et al. 2021). Moreover, high passenger volume was associated with the higher infection rate of destinations (e.g. bars, restaurants, and sport events) (Zhang et al. 2021b) and the least deprived area (Ha et al. 2022). It not only promotes the spread of virus to other parts of the city but also may lead to an outbreak in carriages (Hamidi and Hamidi, 2021). Governments should pay more attention to crowded public destinations around the stations of public transports.

During the pandemic weeks, the number of subway travels in weekdays was 0.3 times higher than that of weekends. However, it is difficult to keep long time on the target of “dynamic zero”(dynamic zero means that preventive measures should be strengthened when cases appear. Under normal circumstances, moderate epidemic prevention measures with *R*_*t*_ =1 or slightly higher than 1 can be maintained) during weekdays on public transports because of large number of necessary travels (e.g. go to work/school). Children took subways more during weekends with most of travels to entertainment areas. The local government should strengthen the Omicron prevention and control on hot subway lines or limit unnecessary travels. In order to achieve the requirement of “dynamic zero “, *R*_*t*_ needs to be controlled smaller than 1 (Guo et al. 2022). If the pandemic is difficult to be controlled in weekdays, the government can slightly reduce the prevention and control requirements during weekdays (*R*_*t*_ slightly higher than 1) and strictly control the pandemic on weekends (*R*_*t*_ < 1) to finally make the average *R*_*t*_ of 1 during the whole week. This optimization method can effectively reduce the cost of the pandemic prevention and control.

Several limitations were existed in the study. Firstly, four population groups were divided according to the types of smartcards, however, as the default card without any discount, adult’s cards may be used by other population groups. Secondly, the determination of each passenger’s route is based on the shortest path method, which assumes the same time spent between stations and neglects differences in routes chosen due to fares, personal habits, etc. Finally, the selected four pandemic weeks may be biased due to other factors, such as the intervention made by the local government. In the future, real close contact behaviors of different types of passengers should be detected to analyze the infection risk via clos contact route in detail. Moreover, the study on virus transmission could be extended from subway to other public transports such as bus and taxi.

## Conclusion

Due to the pandemic, the total number of MTR passengers decreased by an average of 41.0%, and the number of passengers on the same carriage of adults, children, students and senior citizens during weekdays (weekends) decreased by 34.5% (47.8%), 80.0% (78.3%), 72.9% (70.3%), and 30.2% (42.9%), respectively. During the rush hour of pandemic weekdays, the *PSC* of adults, children and students decreased by 32.6%, 88.1% and 81.4%, respectively. Moreover, we found that work from home and staggered shift pattern of workers can reduce the infection risk effectively. However, school suspension is not that effective as we expected due to small number of children/students and relatively high resistance to COVID-19.

## Supplementary Information

**Additional file 1: Figure S1.** Daily number of reported cases in Hong Kong during four pandemic weeks from January 1, 2020 to January 31, 2021. **Figure S2.** The number of new daily cases in Hong Kong from January 23, 2020 to May 31, 2021. **Figure S3.** MTR route map of Hong Kong. **Figure S4.** Number of trains in operation by time. **Figure S5.** The distribution on contact time in the same train of four population groups. (The small block diagram shows the probability distribution when the contact time of the four groups exceeds 100 minutes). **Figure S6.** Probability distribution of daily number of possible repeated passengers on the same subway of four population groups. **Figure S7.** Distribution of daily number of repeated contacts passengers on the same subway of four populations. **Figure S8.** Contact matrix in rush hours. (A) Absolute value; (B) relative value. **Figure S9.** Contact matrix in non-rush hours. (A) Absolute value; (B) relative value. **Figure S10.** The number of passengers in the same carriage (*PSC*) of adults, children and students under work from home or school suspension. **Figure S11.** Daily number of passengers in the same train. **Figure S12.** Change of daily number of passengers on the same train (*DPST*) of four population groups by travel reduction.

## Data Availability

Data cannot be made available for reasons disclosed in the data availability statement.
