# Supplementary material for: Changes on local travel behaviors under travel reduction-related interventions during COVID-19 pandemic: a case study in Hong Kong
Source: City Built Enviro. 2023 Mar 6;1(1):5. doi: 10.1007/s44213-023-00006-z (PMC9985955; doi:10.1007/s44213-023-00006-z)
Supplement: Supplementary file 1 — Additional file 1: Figure S1. Daily number of reported cases in Hong Kong during four pandemic weeks from January 1, 2020 to January 31, 2021. Figure S2. The number of new daily cases in Hong Kong from January 23, 2020 to May 31, 2021. Figure S3. MTR route map of Hong Kong. Figure S4. Number of trains in operation by time. Figure S5. The distribution on contact time in the same train of four population groups. (The small block diagram shows the probability distribution when the contact time of the four groups exceeds 100 minutes). Figure S6. Probability distribution of daily number of possible repeated passengers on the same subway of four population groups. Figure S7. Distribution of daily number of repeated contacts passengers on the same subway of four populations. Figure S8. Contact matrix in rush hours. (A) Absolute value; (B) relative value. Figure S9. Contact matrix in non-rush hours. (A) Absolute value; (B) relative value. Figure S10. The number of passengers in the same carriage (PSC) of adults, children and students under work from home or school suspension. Figure S11. Daily number of passengers in the same train. Figure S12. Change of daily number of passengers on the same train (DPST) of four population groups by travel reduction. [file 44213_2023_6_MOESM1_ESM.docx]

**Supplementary Information**

All interventions for COVID-19 prevention and control in subways considered in this study are listed below:

1. *Work from home (class suspension)*: Firstly, obtained the daily rush hours (morning and evening peaks) of adults (students and children). The difference on number of adult (student and child) passengers between rush hours and the average value of non-rush hours was regarded as the workers (students and children) who need to go to work (schools). When simulation on work from home (school suspension), adult (student and child) commuters was assumed not to take the MTR to the workplace (school).
2. *Staggered shift travel pattern:* Workers (students/children) during rush hours were uniformly assigned into 7:00-10:00 (6:30-9:30) and 17:00-20:00(15:00-18:00).
3. *Reduction on subway riding*: focusing on a population group, if the proportion of their travels is reduced by 10%, it is considered that 10% of passengers of the group would not take the MTR, while local travel behaviors of passengers from other groups would remain unchanged. The travel reduction ratio of each population group was changed from 0% to 90% with an interval of 10% in the simulation.

Other parameters involved in this study were explained as follows:

1. *Daily number of possible repeated contacts on the same train* (*DRC*): if passengers *i* and *j* were on the same subway train *n* times in a day, it means that *i* and *j* had daily repeated contacts of n-1. (From path finding modelling shown in Appendix A, all routes of passengers could be assessed based on the entry and exit station. Based on the schedule of train, the probability of two passengers in the same train could be estimated.)
2. *Frequency of possible repeated contacts on the same train* (*FRC*): the frequency of possible repeated contacts on the same train (min^-1^).
3. *Daily number of passengers on the same train* (*DPST*): the number of passengers who have shared the same train with passenger *i* during the whole day.
4. *Daily duration on the same train*: in the process of taking the subway in a single day, the time that he and passenger *j* spend in the same train is t, then t is called the daily duration on the same train.
5. *Number of passengers* *in the same carriage* (*PSC*): all passengers in the same carriage were regarded as contact with each other.
6. *Contact matrix*: element A (*i*, *j*) represents the daily number of passengers in population group *i* (e.g. adult, child) in the same carriage with passengers in population group *j.*
7. *Relative contact matrix*: matrix $A_{2}$ can be obtained if all elements in contact matrix $A_{2}$ was divided by the total number of passengers who are in in the same carriage with population group *i*, and $A_{2}\left( i,j \right)=\frac{A_{1}\left( i,j \right)}{\sum_{j=1}^{4} A_{1}\left( i,j \right)}$. $A_{2}\left( i,j \right)$ showed the percent of population group *j* in all passengers who is in the same carriage with population group *i*. Relative contact matrix $A_{3}$ can be obtained if all elements in $A_{2}$ was divided by the percentage of passengers with population group *i*, $P_{t}(i)$. The sum of $P_{t}(i)$ of four population groups is 1. $A_{3}\left( i,j \right)=\frac{A_{2}\left( i,j \right)}{P_{t}\left( i \right)}$. $A_{3}\left( i,j \right)$ shows the relative contact rate of population group *i* to *j* in the same carriage (high value showed that *i* tend to contact with *j* in subways).


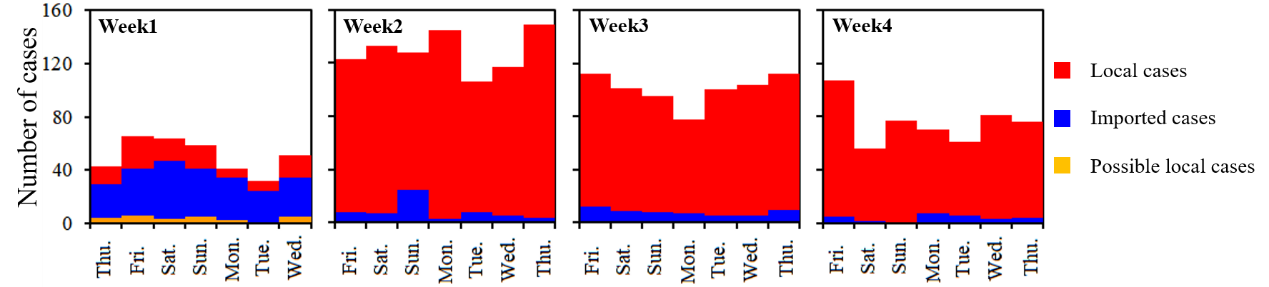


**Figure S1.** Daily number of reported cases in Hong Kong during four pandemic weeks from January 1, 2020 to January 31, 2021.


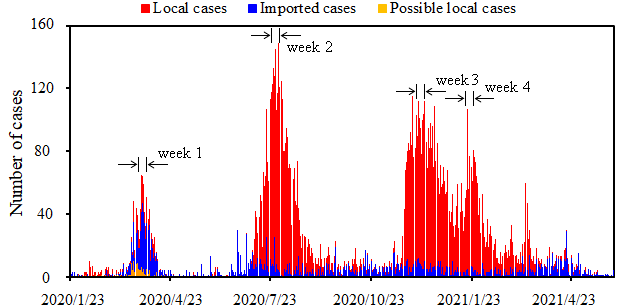


**Figure S2.** The number of new daily cases in Hong Kong from January 23, 2020 to May 31, 2021.


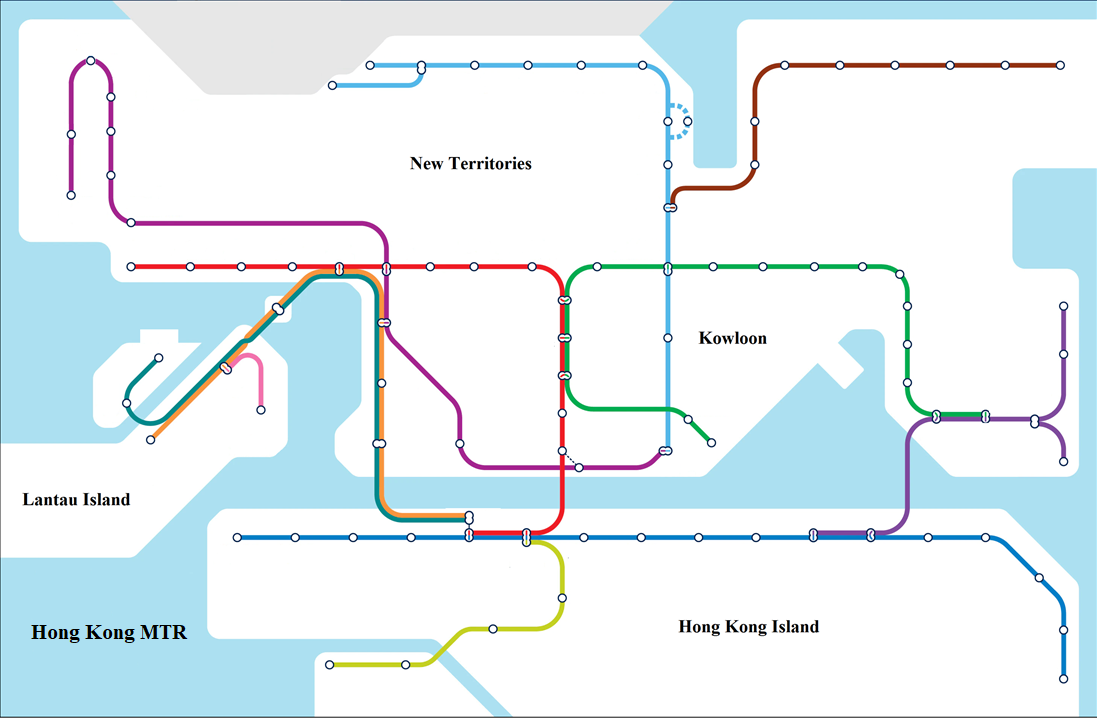


**Figure S3.** MTR route map of Hong Kong


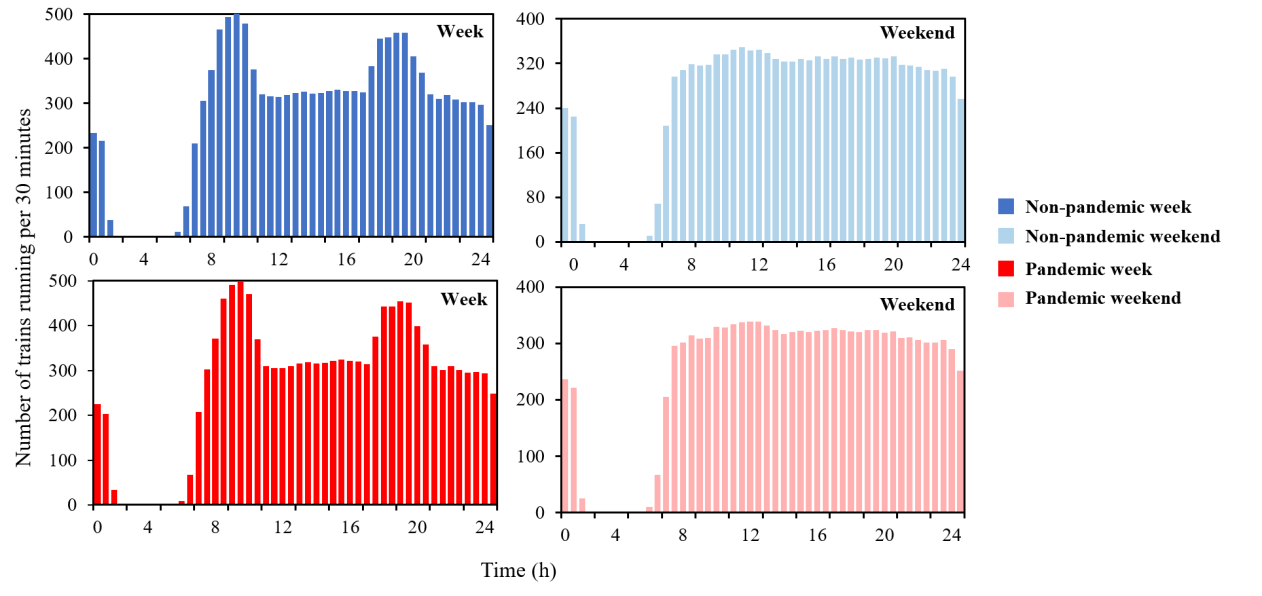


**Figure S4.** Number of trains in operation by time.


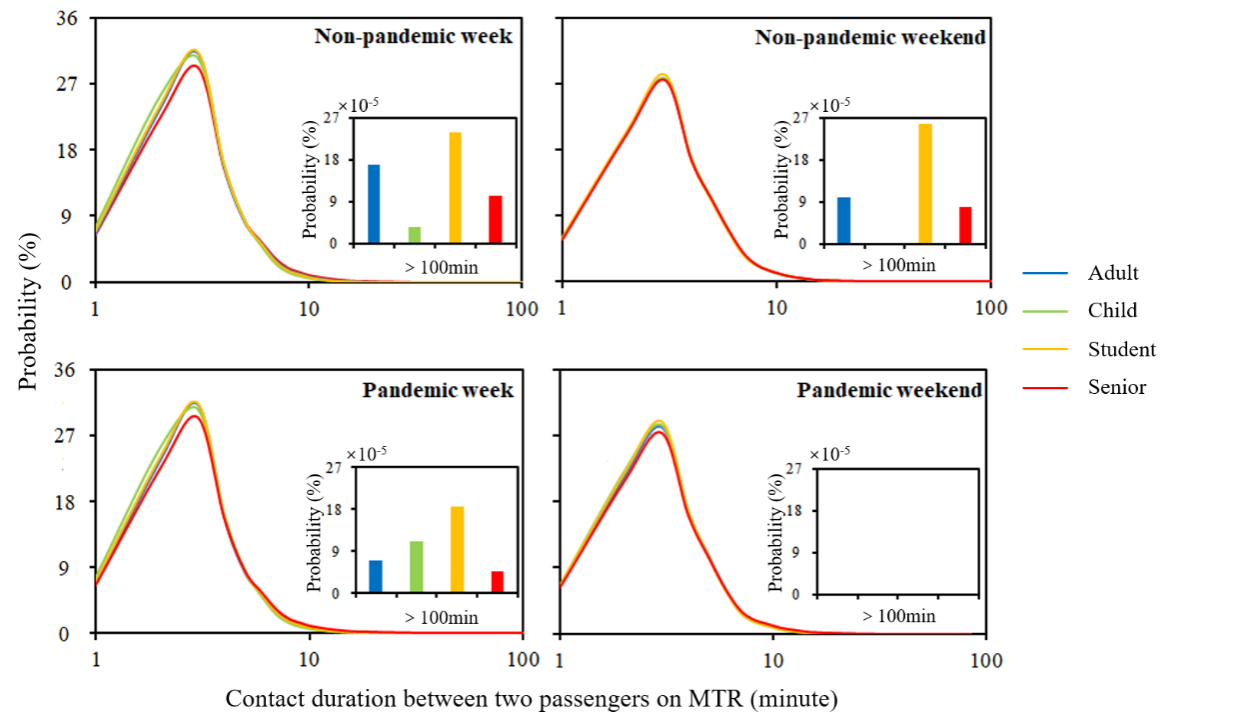


**Figure S5.** The distribution on contact time in the same train of four population groups. (The small block diagram shows the probability distribution when the contact time of the four groups exceeds 100 minutes)


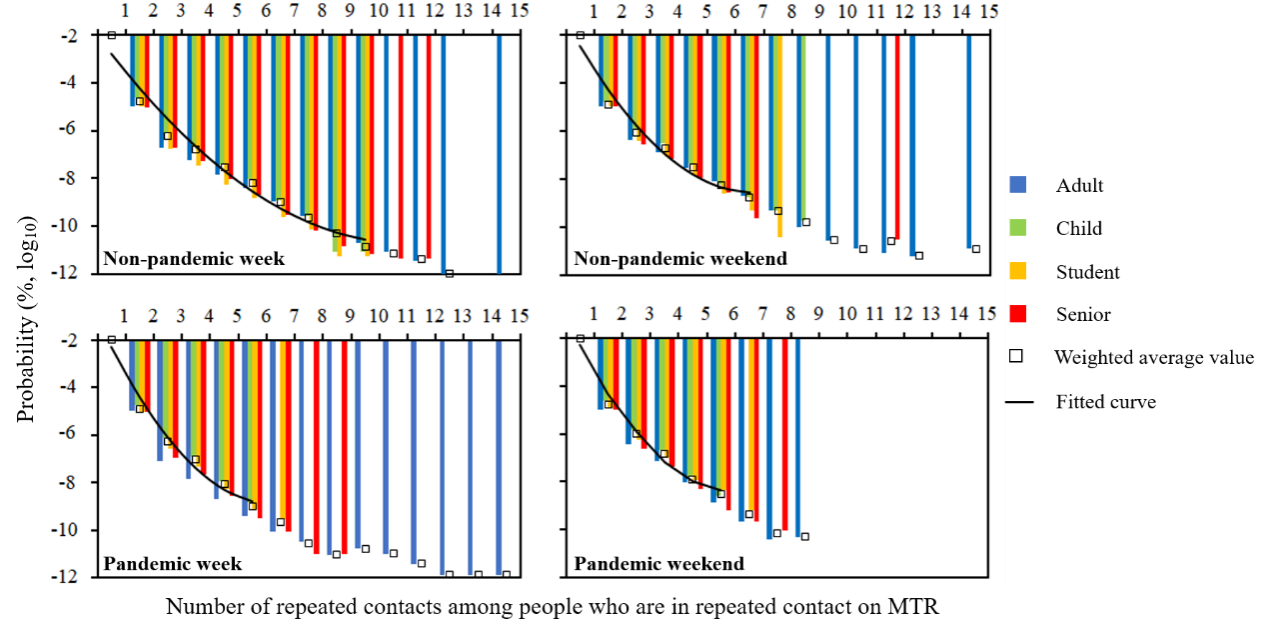


**Figure S6.** Probability distribution of daily number of possible repeated passengers on the same subway of four population groups.


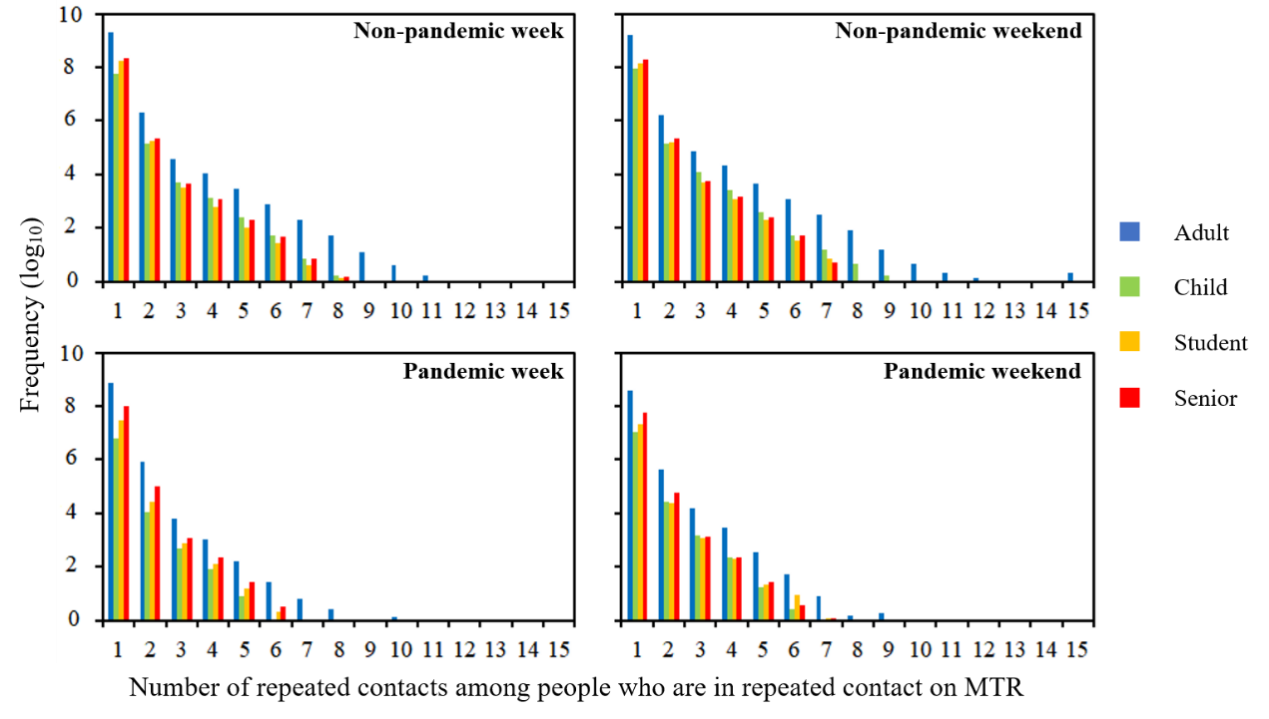


**Figure S7.** Distribution of daily number of repeated contacts passengers on the same subway of four populations


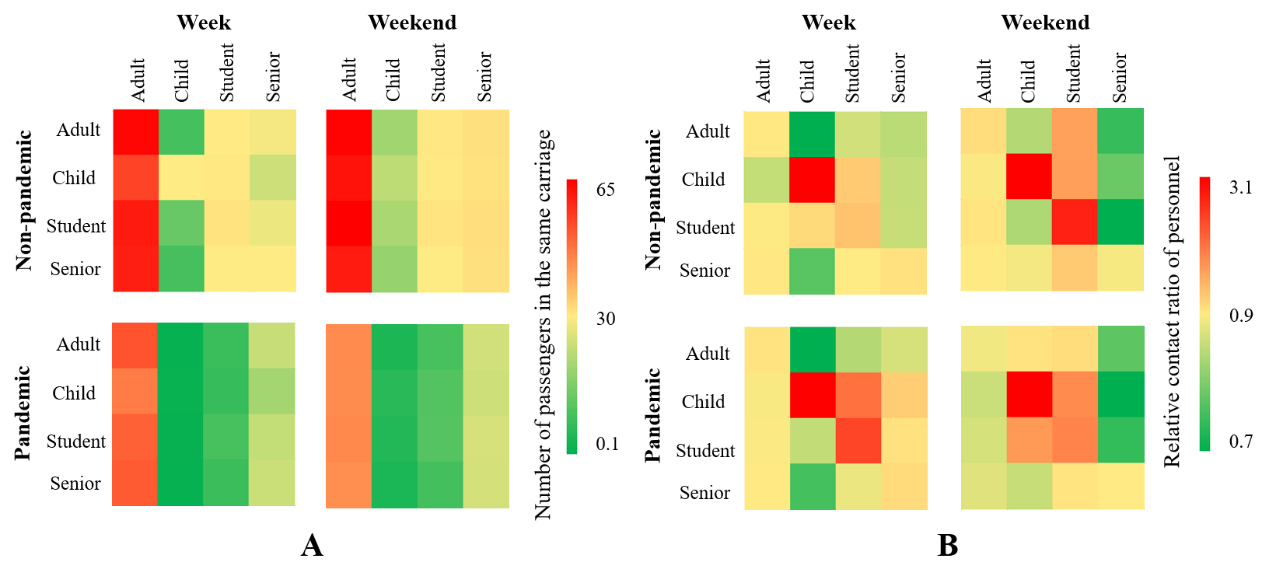


**Figure S8.** Contact matrix in rush hours. (A) Absolute value; (B) relative value.


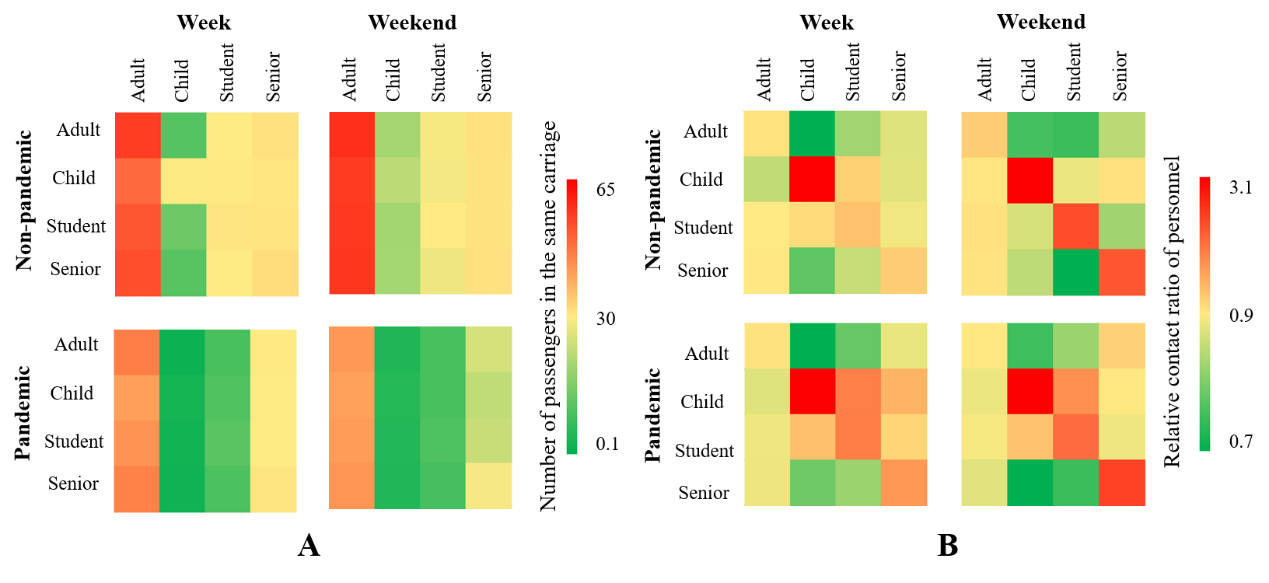


**Figure S9.** Contact matrix in non-rush hours. (A) Absolute value; (B) relative value.


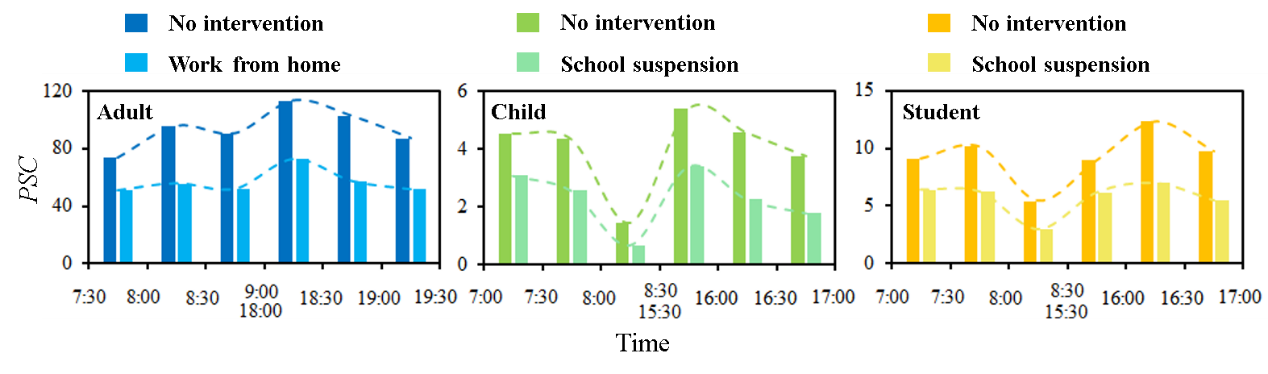


**Figure S10.** The number of passengers in the same carriage (PSC) of adults, children and students under work from home or school suspension.


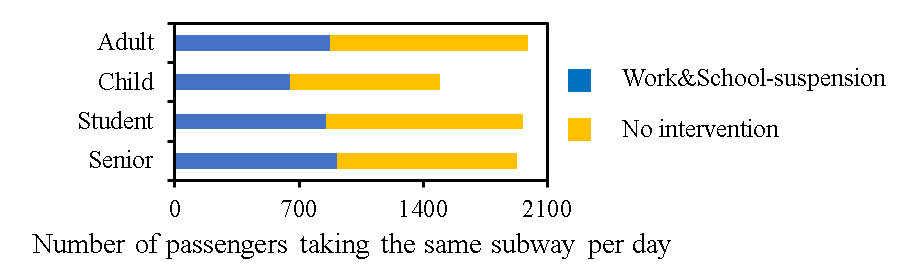


**Figure S11.** Daily number of passengers in the same train


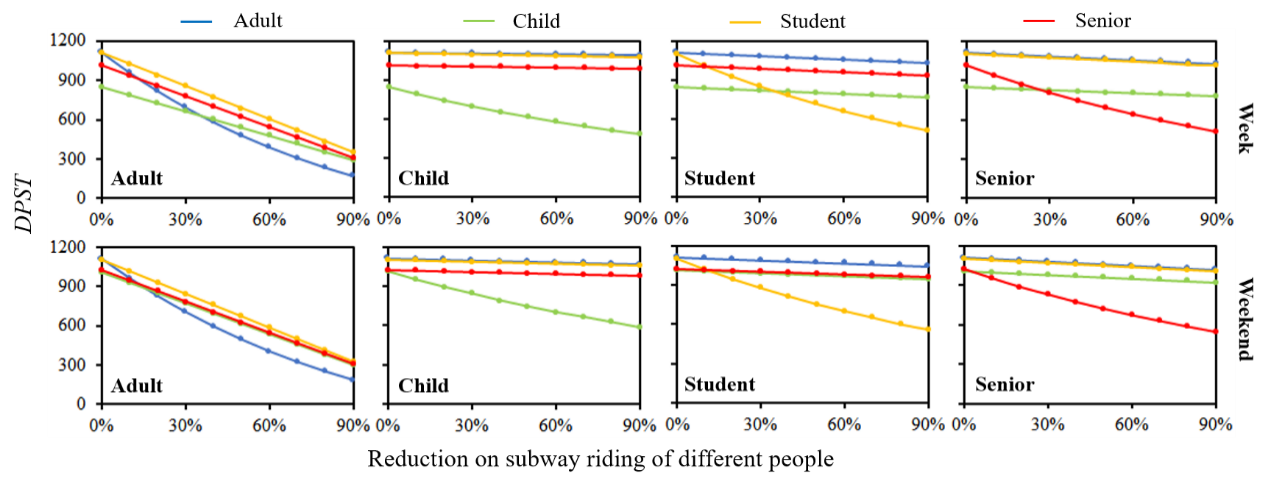


**Figure S12.** Change of daily number of passengers on the same train (*DPST*) of four population groups by travel reduction.
